# The impact of geo-political socio-economic factors on vaccine dissemination trends: a case-study on COVID-19 vaccination strategies

**DOI:** 10.1186/s12889-023-17000-z

**Published:** 2023-11-02

**Authors:** Ritu Chauhan, Gatha Varma, Eiad Yafi, Megat F. Zuhairi

**Affiliations:** 1 Centre for Computational Biology and Bioinformatics, Amity University, Noida, Uttar Pradesh India; 2 Amity Institute of Information Technology, Amity University, Noida, Uttar Pradesh India; 3 Faculty of Engineering and Information Technology, University of Technology Sydney, Sydney, New South Wales Australia; 4 UniKL – LR Univ Joint ICT Laboratory (KLR-JIL), Universiti Kuala Lumpur, Malaysia – La Rochelle University, France, Kuala Lumpur, Malaysia

**Keywords:** COVID-19, Vaccination, Prognosis and diagnosis, Artificial intelligence, Machine learning

## Abstract

**Background:**

The world in recent years has seen a pandemic of global scale. To counter the widespread loss of life and severe repercussions, researchers developed vaccinations at a fast pace to immunize the population. While the vaccines were developed and tested through extensive human trials, historically vaccines have been known to evoke mixed sentiments among the generic demographics. In the proposed study, we aim to reveal the impact of political and socio-economic factors on SARS-Cov-2 vaccination trends observed in two hundred and seventeen countries spread across the six continents.

**Methods:**

The study had hypothesized that the citizens who have lower trust in their government would be less inclined towards vaccination programs. To test this hypothesis, vaccination trends of nations under authoritarian rule were compared against democratic nations. Further, the study was synthesized with Cov-2 vaccination data which was sourced from Our World Data repository, which was sampled among 217 countries spread across the 6 continents. The study was analyzed with exploratory data analysis and proposed with relevance and impacting factor that was considered for vaccine dissemination in comparison with the literacy rate of the nations. Another impacting factor the study focused on for the vaccination dissemination trends was the health expenses of different nations. The study has been synthesized on political and socio-economic factors where the features were ardently study in retrospect of varied socio- economic features which may include country wise literacy rate, overall GDP rate, further we substantiated the work to address the political factors which are discussed as the country status of democratic or having other status.

**Results:**

The comparison of trends showed that dissemination of SARS-Cov-2 vaccines had been comparable between the two-opposing types of governance. The major impact factor behind the wide acceptance of the SARS-Cov-2 vaccine was the expenditure done by a country on healthcare. These nations used a large number of vaccines to administer to their population and the trends showed positive growth. The overall percentage of vaccine utilized by countries in quantitative terms are Pfizer/BioNTech (17.55%), Sputnik V (7.08%), Sinovac (6.98%), Sinopharm/Beijing (10.04%), Oxford/AstraZeneca (19.56%), CanSino (2.85%), Moderna (12.05%), Covaxin (3.28%), JohnsonandJohnson (10.89%), Sputnik Light (3.07%), Novavax (3.49%). While the nations with the lowest healthcare expenses failed to keep up with the demand and depended on vaccines donated by other countries to protect their population.

**Conclusions:**

The analysis revealed strong indicators that the nations which spend more on healthcare were the ones that had the best SARS-Cov-2 vaccination rollout. To further support decision-making in the future, countries should address the trust and sentiment of their citizens towards vaccination. For this, expenses need to be made to develop and promote vaccines and project them as positive health tools.

## Introduction

The global stance, in the current scenario of the pandemic, has imposed a substantial burden on healthcare practitioners. The ultimate aim of researchers and scientists worldwide is to fight the ongoing situation and discover significant vaccines which can reduce the mortality rate among society. As we know, the decisive way to overcome the ongoing situation is to immunize the population. Moreover, the existing circumstances infer that, if we need to prevent and control the spread of COVID-19, then immunization would play an imperative role.

Certainly, COVID-19 has imprudently created several challenges among young researchers, scientists, and healthcare practitioners around the globe [1–4]. To address the key challenges, the interdisciplinary research areas such as education, healthcare, industrial, stock market, food and beverage were examined to determine the factors i.e. socio, behavioral, economical parameters in the vaccine strategy which can directly or indirectly influence the study [2, 3]. Hence, the trial of studies was performed at varying stages which included the clinical trials, spatial parameters of the spread of disease, factors corresponding to disease patients, and others. To discuss the overall impact of studies conducted, the challenge is to disseminate the data and predict information from big databases [5, 6].

To address the vital issues of real-world data complexity, several adoptive technologies such as computer vision, Tele health was need of time and assistive technologies implemented such as visualization-based models to assist the need of the patients also several communication modules were implemented to assist with the work from home and support the overall population to best fit the current scenario [7]. Also, we can say that to curb the spread of COVID 19, Machine learning (ML)and Artificial Intelligence (AI) are well-known techniques, which are deployed to discover hidden information from varied databases. The global network of clinicians, scientists, and healthcare practitioners are trying to accelerate the research programs while utilizing AI and ML to develop an effective and efficient COVID -19 vaccine that can benefit the human population among all age groups [8, 9]. Usually, ML and AI are evolved to develop a prediction-based tool or decision making, which can outline the future adverts of healthcare outcomes [10].

We can say that the healthcare organizations are explicitly searching for appropriate technology which can track the epidemiological synchronization or cofactors which can relate to pandemic spread [9, 10]. However, cofactors can easily studied with AI and ML which can set vulnerable benefits of diagnosis while measuring the effectiveness of the drug, spread of COVID 19, and detecting the co-vital features associated with the disease [11–13]. Further, we can say that AI and ML-based technology are the decision-making platforms which can assist healthcare practitioners to fight the battle of COVID -19 while giving a recommendation, medical features extractions, patient-assisted control, and other services which we can think of.

Further, the utilization of ML and AI has flourished their advances in the pandemic era, victoriously in several application domains which include military organizations, healthcare, business analytics, and other technological intervention while gaining momentum and success stories [11–13]. For example, the chest CT scan images were applied with medical imaging analysis using ML and AI-based algorithms to detect the prognosis of the disease in a short period, as well as citing the drug discovery module which can benefit the patient [14, 15]. In addition, ML and AI-based studies were applied to study the societal and behavioral implications of patients and develop intelligent models which can benefit the post-Covid era of patients [16]. Moreover, several human transmission models were also generated to identify and predict the outbreak of disease and other severities aligned with spread [16, 17].

Further, considering the relative global impact of vaccination with vital features such as literacy rate, disabilities, older generation and many other factors in retrospect of the citizens and certain formulation suggested that users those have lower trust in their government were less inclined towards vaccination programs [17]. In accordance, to the previous work we tried to determine the vaccination trends among varied nations under authoritarian rule as well as comparison was strategized against democratic nations [17, 18]. The comparison of trends showed that dissemination of the SARS-Cov-2 vaccine had been comparable between the two-opposing types of governance. Further, the study was proposed with relevance and impacting factor that was considered for vaccine dissemination in comparison with the literacy rate of the nations. The correlated factor was referred to as the country's literacy rate, which is an indicator of its economy and living standards [14]. In agreement with the above problem, the vaccination trends were represented, with vital features of lesser literacy rates having extremely low and flat growth curves for vaccination numbers [15]. In similar, another impacting factor that the study focused on was the vaccination dissemination trends in comparison with the health expenses of different nations. The curves were strong indicators that the nations that spend more on healthcare were the ones that had the best SARS-Cov-2 vaccination rollout [16]. These nations used a large number of vaccines to administer to their population and the trends were positive growth [17]. While the nations with the lowest healthcare expenses failed to keep up with the demand and depended on vaccines donated by other countries to protect their population [18].

In the proposed study, we aim to reveal the impact of political and socio-economic factors on SARS-Cov-2 vaccination trends in different countries. The study has been synthesized on political and socio-economic factors where the features were ardently study in retrospect of varied socio- economic features which may include country wise literacy rate, overall GDP rate, further we substantiated the work to address the political factors which are discussed as the country status of democratic or having other status which are discussed elaborated in methodology section.

### Literature review

Data science can be discussed as a vibrant tool for developing and supporting vaccine dissemination. The major role is to discover the protein structure which can enable the clinicians and data scientists to discover the patterns for future research repositories and identification of drug discovery. We can say that vaccine is the only possibility that can control the dissemination of COVID 19 cases around the globe [18]. However, the statistics represents that few vaccines have received permission to use, as in case of emergency conditions that can benefit the healthcare practitioners and others. In context, several vaccination programs are laid into the process to assure the wellbeing, safety, and control of the blowout of the COVID-19 [18–20]. Hence, we can say that earlier vaccine development was a trivial task, but the advent technology introduced has the ability to identify the protein structure and develop a new drug for a deeper understanding of the virus. Google with integrated technology of AI launched the Alphafold tool, which tends to be an automatic and specialized tool that can predict the new vital 3D protein structure while input as the genetic sequence [21]. The above cited approached focused on detection of the untreated proteins which regulate the SARS-COV-2 virus and generate a protein structure [17, 22].

The concept of reverse vaccinology (RV) with ML tools was utilized to forecast and develop a vaccine for COVID -19. The RV was applied to discover the pathogen using genomes. The study prevailed to discover the proteins which were responsible for the virus which includes SARS-CoV, MERS-CoV, HCoV-229E, and others where were discovered from Uniprot proteins. Moreover, they have exploited the ML techniques Vaxign-ML to detect and discover the signal behavior of the proteome of the virus, to forecast the relevance of biological signals [23]. Further, the Vaxign-ML model was formulated using classifier Random Forest, structural and vector proximity, the context of modeling which was based on ML and RC, was applied to determine the protein and its correspondence level [23].

In similar, the control and spread of any communicable disease depend upon the vaccine instantiated. Certainly, the development of these vaccines is not an easy task, moreover, several challenges and barriers exist to determine the effective and efficient drug which can handle the generic data. In the past several studies are introduced by researchers and health care practitioners to study the overall effect of the vaccine [17–22]. Comparably, a study was proposed to discuss the encounters of the vaccine in context with structural and attitudinal patterns in the US population [24, 25]. Where the structural barriers were discussed as the service which was not accessible such as transportation services, or going out and acquiring public services. Further, attitudinal barriers refer to the fact the beliefs which a person acquires the fear of communicable disease or his unwillingness to accept the facts due to perception of the mind. Moreover, in the context of vaccination, it may be discussed as the barrier which develops as the aftermaths or present vaccination programs and determines the risks associated with same. Similar outcomes can be controlled by several recommendation programs, where the effectiveness and importance of COVID- 19 can help them reduce the barriers among the population, which an individual acquires due to rational information [24, 25].

## Method and materials

COVID 19 has substantially worn the world upside down which has created several inhibitions to detect hidden patterns and information from large scale databases. However, the current study of approach focuses on vaccine dissemination patterns around the globe. The study was based on databases generated from public domain repository of United Nations World Population Prospect, where the datasets compromise of subnational locations such as England, Northern Ireland, Scotland, Wales and others [26]. Also, the overall estimation was calculated on the basis of total vaccinations conducted with doses administrated at each level in correspondence people fully vaccinated with per hundred million. The dataset was analyzed to measure the potential factors in context with impact of political and socio-economic factors on SARS-Cov-2 vaccination trends in different countries. The study has been synthesized on political and socio-economic factors where the features were ardently study in retrospect of varied socio- economic features which may include country wise literacy rate, overall GDP rate [27, 28]. Further, we have utilized the secondary data extracted to explore the international trends of vaccination. The current study of approach was focused to provide appropriate geographical coverage of vaccination with stratified sampling and exploratory data analysis.

### Model

The exploratory data analysis (EDA) is conducted among the databases to detect hidden patterns, information and knowledge from the data. The data investigated was preprocessed for null values, missing values and inconsistent values to measure the exploratory features summarization. The proposed study was implemented using python 3.9.0 version using NumPy package, Matplotlib library and pandas to explore the complex patterns among the data. Further, data was explored with graphs and distribution curves to synthesize and identify the potential outlines in the data. The analysis also included varied statistics such as mean, median, standard deviation for varied features in the datasets.

Additionally, the study was categorized with relevance and impacting factor which were considered for vaccine dissemination in comparison with the literacy rate of the nations. Also. Target features were considered for the vaccination dissemination trends for calculating the health expenses of different nations. The study has been synthesized on political and socio-economic factors where the features were ardently study in retrospect of varied socio- economic features which may include country wise literacy rate, overall GDP rate, further we substantiated the work to address the political factors which are discussed as the country status of democratic or having other status.

### Descriptive analysis

Vaccines have been one of the biggest preventive measures that were developed over the past century to tackle dreaded diseases. However, the major diseases which tends to cured with vaccination is smallpox and rinderpest which has substantially outcasted the population with their rendered threats. While Covid-19 vaccinations have once again raised the debate about a population's trust in various aspects of this process, studies were done before the pandemic also helped in understanding the factors that drive acceptance rates [29, 30].

A study done by Larson et al. in 2016 [17] had analyzed trust in vaccinations in terms of four factors, namely its importance, its safety, its effectiveness, and religious compatibility. Respondents from sixty-seven countries were surveyed for their confidence in vaccines. The survey gauged sentiment on a five-point Likert scale, where answers were discrete values of strongly agree, tend to agree, do not know, tend to disagree, strongly disagree. After the removal of the neutral answer of 'do not know', the countries were ranked based on low confidence. The countries that showed the most negative sentiment towards vaccination before the SARS-Cov-2 pandemic in terms of the four different factors are listed in Table 1.

**Table 1:** The top ten countries that displayed the least trust in vaccinations before the SARS-Cov-2 pandemic

Rank	Vaccine Safety	Vaccine Importance	Vaccine Effectiveness	Religious Compatibility
1	France	Russia	Bosnia and Herzegovina	Mongolia
2	Bosnia and Herzegovina	Italy	Russia	Thailand
3	Russia	Azerbaijan	Italy	Mexico
4	Mongolia	China	France	China
5	Greece	Slovenia	Greece	Vietnam
6	Japan	Hong Kong	Slovenia	Bangladesh
7	Ukraine	Ukraine	Romania	Panama
8	Iran	Mexico	Ukraine	Kosovo
9	Slovenia	Greece	Latvia	India
10	Armenia	France	Serbia	Fiji

As can be seen from the rankings, European and Western Pacific nations were least confident of vaccinations. Developed nations like Japan and France were unexpected entries. The distrust of the population of France can be attributed to the vaccine-related controversies that plagued the nation in the past two decades [31]. Japan is known to be a risk-averse nation and therefore the population shows a major concern in vaccine safety to fully trust them [32]. Table 2 lists the top ten countries that displayed positive sentiment towards vaccines.

**Table 2:** The top ten countries that displayed the most trust in vaccinations before the SARS-Cov-2 pandemic

Rank	Vaccine Safety	Vaccine Importance	Vaccine Effectiveness	Religious Compatibility
1	Finland	Kosovo	Australia	Argentina
2	Algeria	Iceland	Tunisia	Bulgaria
3	Ethiopia	Ethiopia	Denmark	Portugal
4	Portugal	Brazil	Finland	Sweden
5	Indonesia	Vietnam	Saudi	Morocco
6	Ecuador	Philippines	Iceland	Armenia
7	Philippines	Argentina	Philippines	Australia
8	Saudi Arabia	Ecuador	Ethiopia	Brazil
9	Argentina	Iran	Ecuador	Saudi Arabia
10	Bangladesh	Bangladesh	Argentina	Finland

While the countries with positive sentiment towards vaccination are a mix of developed and developing nations, the latter were more accepting. It should also be noted that religious fundamentalism might impede vaccine acceptance, but it cannot be linked to a certain faith type, as shown by the presence of Saudi Arabia which has a majorly Muslim population [33].

The SARS-Cov-2 vaccination data was sourced from Our World in Data repository hosted on Github [22]. This dataset is comprised of two hundred and seventeen countries spread across the six continents. Table 3 lists the attributes of the data that was accessed till July 2021.

**Table 3:** The attributes of the vaccination data sourced from Our World in Data repository

Attribute Name	Description
location	Name of the country or a geographical region
iso_code	Three letter country code
date	date of the observation
total_vaccinations	total number of doses administered
total_vaccinations_per_hundred	total number of doses administered per hundred persons
daily_vaccinations_raw	daily change in the total number of doses administered
daily_vaccinations	new doses administered per day smoothened over seven days
daily_vaccinations_per_million	new doses administered per day smoothened over seven days per 1,000,000 persons
people_vaccinated	total number of people who received at least one vaccine dose
people_vaccinated_per_hundred	total number of people who received at least one vaccine dose per hundred persons
people_fully_vaccinated	a total number of people who received all doses prescribed by the vaccination protocol. Includes both doses of vaccine, if applicable
people_fully_vaccinated_per_hundred	the total number of people who received all doses prescribed by the vaccination protocol per hundred persons. Includes both doses of vaccine, if applicable

The data repository also contained information on Covid vaccines that were administered till the time of this study. These were Oxford/AstraZeneca, Pfizer/BioNTech, Sinopharm/Beijing, Sinovac, SputnikV, JohnsonandJohnson, Moderna, Covaxin, CanSino, Sinopharm/Wuhan, Abdala, Soberana02, QazVac, Sinopharm/HayatVax, EpiVacCorona, RBD-Dimer [22]. Their usage breakdown is shown in Table 4. The three vaccines that have been administered in the majority are Oxford/AstraZeneca, Pfizer/BioNTech, and Moderna.

**Table 4:** The percentages breakdown of SARS-Cov-2 vaccines used till July 2021 across the world

Vaccination	Usage
Pfizer/BioNTech:	17.55%
Sputnik V:	7.08%
Sinovac:	6.98%
Sinopharm/Beijing:	10.04%
Oxford/AstraZeneca	19.56%
CanSino:	2.85%
Moderna:	12.05%
Covaxin:	3.28%
JohnsonandJohnson:	10.89%
Sputnik Light:	3.07%
Novavax:	3.49%

Our analysis also aimed to discern the effect of a nation's political regime on the SARS-Cov2 vaccination dissemination. While Authoritarian governments are associated with strict implementation of policies among the residents, democracies offer a choice to its residents. The said choice may take stricter forms in case of emergencies like a pandemic. Therefore, some democracies also saw strict regulations regarding the SARS-Cov2 vaccination dissemination. Nonetheless, we combined the political regimes of different nations [26] for this particular analysis. The dataset contained political regime values as Polity 2 Measure ranges from -10, which corresponded to autocracy, to + 10 for full democracy. For the ease of analysis, the Polity indices were categorized as Authoritarian where the measure ranged between -10 to -6, Limited Authoritarian for Polity measures of -5 to -1, Limited Democracy for 0 to 5, None for non-specified values, and finally Democracy for values 5 to 10. The conversion helped in converting the feature to a categorical distribution.

## Results and discussion

**Fig. 1:**
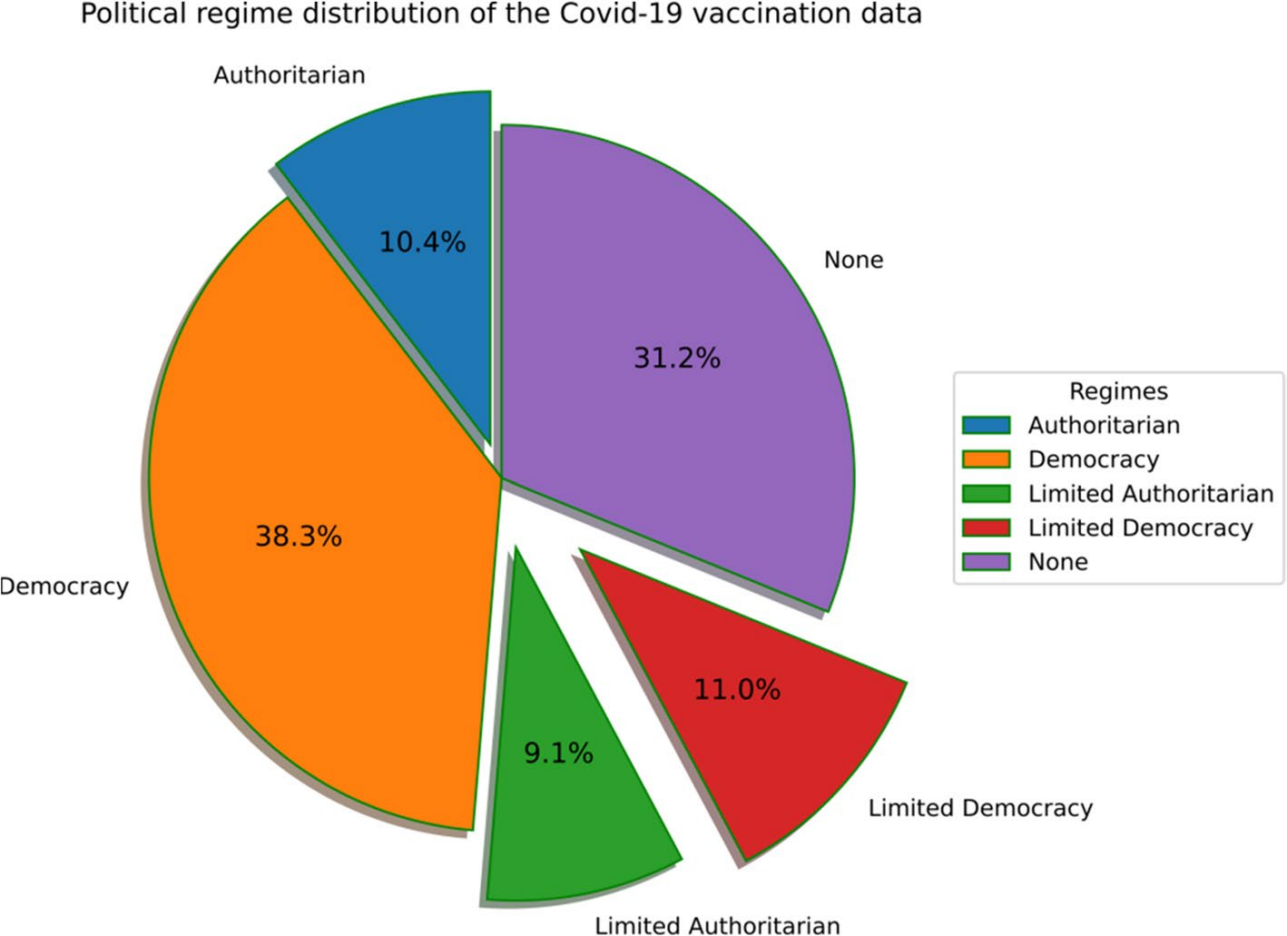
The percentages breakdown of political regimes for countries that were surveyed for SARS-Cov-2 vaccination

The proposed analysis aimed to reveal the impact of political and socio-economic factors on SARS-Cov-2 vaccination trends in different countries. The political regimes of different nations were fetched from Our World in Data repository [26] to discern the distribution of regimes and the vaccination trends. The dataset contained political regime values as Polity 2 Measure ranges from -10, which corresponded to autocracy, to + 10 for full democracy. For ease of analysis, the Polity indices were categorized as Authoritarian where the measure ranged between -10 to -6, Limited Authoritarian for Polity measures of -5 to -1, Limited Democracy for 0 to 5, None for non-specified values, and finally Democracy for values 5 to 10. Additionally, Authoritarian rule means that the people have to obey the rule and regulations formed by the single political leader. In this rule, the government doesn’t have an established system where the powers can be transformed and the liberal rights can be granted to the people. So, the authoritarian rule confers to the fact that no democracy is granted and hence the people have to subsidies on the rules created by the leader. A limited Authoritarian government is the one where the legalized forces are restricted and obey the rules of authorities. Also, the laws and their restriction on individuals and business are controlled with fewer restriction. Hence, difference between limited authoritarian and authoritarian is constitutional powers are limited in case of limited authoritarian whereas all the powers are controlled by single political leader in case of authoritarian. The democratic rule implies that the power which is elected by the people. It means the people will elect their own minister and hence govern themselves in indirect way. So, democratic way of rule is for the people, by the people and of the people to maintain the freedom at each level. Limited democratic rule can be discussed as the power which restricts the leaders to have absolute power with the inclination towards the lawmakers to abide by the absolute powers created by the government. Also, limited powers are gained by the high-profile individuals and also citizens are required to adhere with the constitutional powers. Hence, Fig. 1 shows the distribution of the five types of political regimes among the vaccination data and democracy emerged as the most common form of governance.

**Fig. 2:**
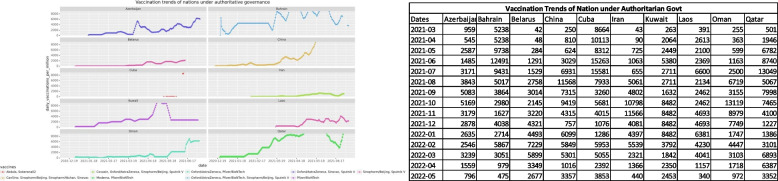
The vaccines used and vaccination trends of nations under authoritarian rule

**Fig. 3:**
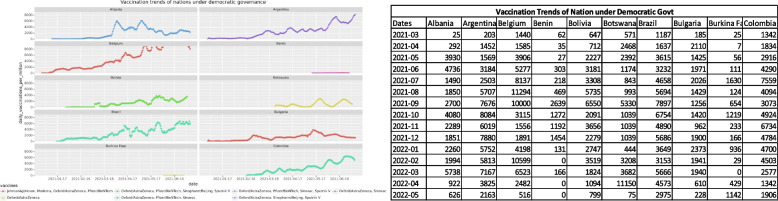
The vaccines used and vaccination trends of some democratic nations

In, Figs. 2 and 3 the illustrated images identify the vaccination trends and vaccines used by the compared nations for varied authoritative and democratic governances.

The comparison of trends shows that dissemination of the SARS-Cov-2 vaccine has been done between the two opposing types of governance. Both types have used a combination of vaccines and the graphs show a rising trend thus marking extensive acceptance among the citizens.

**Fig. 4:**
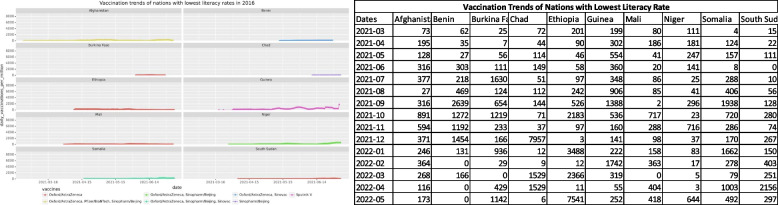
Vaccination trends with the lowest literacy rates in the year 2016

**Fig. 5:**
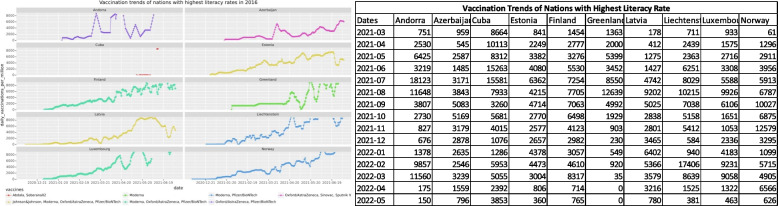
The vaccines used and vaccination trends of nations with the highest literacy rates in the year 2016

The next impacting factor that was considered for vaccine dissemination was the literacy of the nations. The WHO literacy rates from the year 2016 [27] were used to shortlist nations with the lowest and highest literacy rates. The shortlisted nations were then analyzed for preferred vaccines and dissemination trends. Figures 4 and 5 show trends for nations with the lowest and highest literacy rates respectively.

A country's literacy rate is an indicator of its economy and living standards. In agreement with this statement, the vaccination trends show that nations with the poorest literacy rates have extremely low and flat growth curves for vaccination numbers. On the other hand, the nations that have the highest literacy rates boast of good living conditions and better vaccination figures. These nations also used a combination of multiple vaccines for the faster safeguard of their population.

**Fig. 6:**
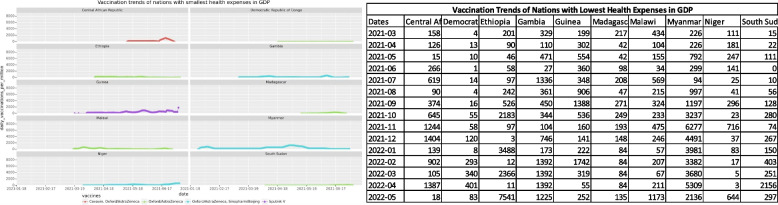
The vaccines used and vaccination trends of nations with the lowest expenditure on healthcare

**Fig. 7:**
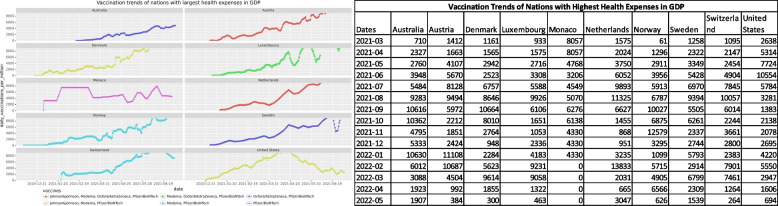
The vaccines used and vaccination trends of nations with the highest expenditure on healthcare

Another impacting factor that this study focused on was the vaccination dissemination trends in comparison with the health expenses of different nations. Each nation sets aside a percentage of the GDP towards the betterment of healthcare and its implementation. The health expenditure data hosted by the World Bank for the year 2018 [28] was used to get expenditure figures of the nations. Figures 6 and 7 show trends for nations with the lowest and highest healthcare expenses respectively. The curves are strong indicators that the nations that spend more on healthcare were the ones that had the best SARS-Cov-2 vaccination rollout. These nations used a large number of vaccines to administer to their population and the trends were positive growth. While the nations with the lowest healthcare expenses failed to keep up with the demand and depended on vaccines donated by other countries to protect their population. The curves grew slowly over time, while some were flat and showed slow progress.

## Conclusion

The human race has progressed through several inventions, and vaccines have been one of the most important of them all.

While vaccines have been successful in improving the life expectancy of the masses, the technology is still riddled with distrust and negative sentiment among the masses. This sentiment gets further aggravated due to controversies and low confidence in scientific research. It is but evident that the difference in ideology can be attributed to the demographics that the persons with negative sentiment belong to. The ideology of a person is governed by their cultural background and the other conditions that prevail in their country. These may include political factors like the government regime, economic factors like GDP and generic economic status, and literacy rates.

In this paper, we have analysed how vaccination dissemination and acceptance were affected by the political and socio-economic factors where the features were ardently study in retrospect of varied socio- economic features which may include country wise literacy rate, overall GDP rate and others. While, the world is battling with the global pandemic of SARS-Cov-2, sentiment towards vaccination has again come into the limelight. The authors have analyzed the confidence in vaccinations before the Covid pandemic happened and found that the European and Western Pacific nations were least confident. Japan, known to be a risk-averse nation also showed a major concern in vaccine safety to fully trust them. The countries with positive sentiment towards vaccination were a mix of developed and developing nations. In the post-Covid scenario, the analysis showed that the dissemination of the SARS-Cov-2 vaccine had been comparable between the authoritarian and democratic types of governance. The vaccination trends also showed that the nations with the poorest literacy rates had extremely low and flat growth curves for vaccination numbers. The nations that spent more on healthcare were the ones that had the best SARS-Cov-2 vaccination rollout. These nations used a large number of vaccines to administer to their population and the trends were positive growth. While the nations with the lowest healthcare expenses failed to keep up with the demand and depended on vaccines donated by other countries to protect their population. With this analysis, the authors hope that better vaccination strategies can be drafted as suited to the geopolitical factors of different countries.

## Data Availability

The qualitative data extracted and analysed during the current study is publicly available but can be discussed or made available from the corresponding author on reasonable request. All documents analysed are publicly available and referenced in this article.
